# Electrophysiological properties of anion exchangers in the luminal membrane of guinea pig pancreatic duct cells

**DOI:** 10.1007/s00424-018-2116-1

**Published:** 2018-02-04

**Authors:** N. Andharia, M. Hayashi, H. Matsuda

**Affiliations:** Department of Physiology, Kansai Medical University, 2-5-1 Shinmachi, Hirakata, 573-1010 Japan

**Keywords:** Bicarbonate, Duct, Exchanger, Pancreas, Patch-clamp, SLC26

## Abstract

The pancreatic duct epithelium secretes the HCO_3_^−^-rich pancreatic juice. The HCO_3_^−^ transport across the luminal membrane has been proposed to be mediated by SLC26A Cl^−^–HCO_3_^−^ exchangers. To examine the electrophysiological properties of Cl^−^–HCO_3_^−^ exchangers, we directly measured HCO_3_^−^ conductance in the luminal membrane of the interlobular pancreatic duct cells from guinea pigs using an inside-out patch-clamp technique. Intracellular HCO_3_^−^ increased the HCO_3_^−^ conductance with a half-maximal effective concentration value of approximately 30 mM. The selectivity sequence based on permeability ratios was SCN^−^ (1.4) > Cl^−^ (1.2) = gluconate (1.1) = I^−^ (1.1) = HCO_3_^−^ (1.0) > methanesulfonate (0.6). The sequence of the relative conductance was HCO_3_^−^ (1.0) > SCN^−^ (0.7) = I^−^ (0.7) > Cl^−^ (0.5) = gluconate (0.4) > methanesulfonate (0.2). The current dependent on intracellular HCO_3_^−^ was reduced by replacement of extracellular Cl^−^ with gluconate or by H_2_DIDS, an inhibitor of Cl^−^–HCO_3_^−^ exchangers. RT-PCR analysis revealed that the interlobular and main ducts expressed all SLC26A family members except *Slc26a5* and *Slc26a8*. SLC26A1, SLC26A4, SLC26A6, and SLC26A10 were found to be localized to the luminal membrane of the guinea pig pancreatic duct by immunohistochemistry. These results demonstrate that these SLC26A Cl^−^–HCO_3_^−^ exchangers may mediate the electrogenic HCO_3_^−^ transport through the luminal membrane and may be involved in pancreatic secretion in guinea pig ducts.

## Introduction

The pancreas plays a pivotal role in digestion. Pancreatic acini secrete digestive enzyme-rich neutral fluid that is not dependent on the presence of the CO_2_/HCO_3_^−^-buffer system. However, ducts secrete a HCO_3_^−^-rich fluid, which is dependent on the presence of CO_2_/HCO_3_^−^-buffer, and that neutralizes acid chyme in the duodenum [32]. The generally accepted model for HCO_3_^−^ transport involves Cl^−^–HCO_3_^−^ exchangers that operate in parallel with cAMP-activated Cl^−^ channels [cystic fibrosis transmembrane conductance regulator (CFTR)] and Ca^2+^-activated Cl^−^ channels, such as TMEM16A/ANO1, on the luminal membranes of duct cells [42, 49]. TMEM16A/ANO1 is also found specifically in the apical membranes of the acinar cells and is the critical channel for the control of acinar fluid secretion [33]. In addition, H^+^–K^+^ pumps and K^+^ channels are expressed on the luminal membrane of pancreatic ducts [11, 28, 45]. K^+^ channels are important for setting the resting membrane potential and for providing the driving force for anion transport, and may provide the transport partners for H^+^–K^+^ pumps [10].

Electrophysiological studies have found a luminal Cl^−^ conductance in rat pancreatic ducts [6, 27]. Single-channel recordings revealed small-conductance Cl^−^ channels on the luminal membrane of duct cells, which were identified as CFTR Cl^−^ channels [6, 7]. The HCO_3_^−^/Cl^−^ permeability ratios of CFTR Cl^−^ channels have been reported as 0.1 to 0.4 [7, 29] and demonstrated to be increased to 1.0 by reducing the intracellular Cl^−^ concentration in pancreatic duct cells [31]. Measurement of intracellular pH and membrane potential of guinea pig duct cells suggested that CFTR Cl^−^ channels provide a significant pathway for HCO_3_^−^ secretion [17].

Another pathway for HCO_3_^−^ secretion across the luminal membrane is the Cl^−^–HCO_3_^−^ exchanger, which has been identified as solute carrier family 26 member A6 (SLC26A6) [16, 20, 48]. SLC26A6 has been found to be electrogenic with a 1Cl^−^/2HCO_3_^−^ exchange stoichiometry in *Xenopus* oocytes and HEK 293 cells [19, 38]. Consistently with this, deletion of *Slc26a6* altered the overall stoichiometry of apical Cl^−^–HCO_3_^−^ exchange in native mouse interlobular ducts, suggesting the upregulation of a Cl^−^–HCO_3_^−^ exchanger with different stoichiometry [41]. Previous studies have demonstrated a functional coupling between CFTR Cl^−^ channels and Cl^−^–HCO_3_^−^ exchange activity in isolated pancreatic interlobular ducts [15, 43]. Furthermore, a computational model suggested that the HCO_3_^−^/Cl^−^ permeability ratio of apical Cl^−^ channels of 0.4 was able to support HCO_3_^−^ secretion [50]. However, few studies have examined the electrophysiological properties and regulation of HCO_3_^−^ conductance across the luminal membrane of native pancreatic duct cells.

The aim of the present study was to identify HCO_3_^−^ conductance that is important for pancreatic secretion. For this purpose, we directly measured HCO_3_^−^ currents through the luminal membrane of guinea pig pancreatic duct cells using the patch-clamp method in the inside-out configuration. We demonstrated that the inward conductance is dependent on intracellular HCO_3_^−^ and extracellular Cl^−^, and is blocked by H_2_DIDS, an inhibitor of anion transporters, and thus conclude that such inward conductance is carried out via anion exchangers on the luminal membrane. Furthermore, we report the expression and localization of the SLC26A family in the interlobular and main pancreatic duct using molecular biological and immunohistochemical analyses.

## Methods

### Preparation of pancreatic duct cells from guinea pigs

Female Hartley guinea pigs (240–450 g, *n* = 35) were sacrificed by carbon dioxide stunning in accordance with protocols approved by the Animal Experimentation Committee, Kansai Medical University. Pancreatic ducts were isolated by enzymatic digestion and microdissection from the pancreas as previously described [12]. The pancreas was removed, and digested with collagenase (Type IV, 124 U/ml; Worthington) and trypsin inhibitor (0.01%; Sigma) in Tyrode solution at 37 °C for 1 h with vigorous shaking. Tyrode solution contained the following (in mM): 140 NaCl, 0.33 NaH_2_PO_4_, 5.4 KCl, 1.8 CaCl_2_, 0.5 MgCl_2_, 5 HEPES, and 5.5 D-glucose; pH was adjusted to 7.4 with NaOH. Interlobular and intralobular ducts (outside diameter of 30–60 μm) were microdissected under a stereomicroscope. The ducts were washed in Tyrode solution and then placed on coverslips pretreated with Cell-Tak (BD Biosciences). In order to allow patch-clamp access to the luminal membranes of the lining of epithelial cells, the ducts were split open by patch pipettes.

### Patch-clamp recording

Standard patch-clamp techniques were used. Patch pipettes, pulled from capillaries of hard borosilicate glass (G-1.5; Narishige), had a resistance of 5–7 MΩ when filled with a standard *N*-methyl-D-glucamine (NMDG)-Cl solution. The standard NMDG-Cl solution contained the following (in mM): 130 NMDG, 130 HCl, 5 EGTA, and 10 HEPES; pH was adjusted to 7.4 with NMDG. The stripped duct was bathed in a standard bicarbonate solution consisting of the following (in mM): 115 NaCl, 5 KCl, 1 CaCl_2_, 1 MgCl_2_, 25 NaHCO_3_, 10 HEPES (pH 7.4, adjusted with NaOH), and 5.5 D-glucose. The standard bicarbonate solution was equilibrated with 5% CO_2_ in O_2_. The membrane potential was corrected for the liquid junction potential at the tip of the patch pipette in the bathing solution, and for that at the tip of the indifferent reference electrode filled with Tyrode solution and placed in the bath. Experiments were conducted at 23–30 °C. After the inside-out configuration was established, the solution in the perfusion chamber was switched to control bicarbonate solution. The control bicarbonate solution contained the following (in mM): 130 KHCO_3_, 5 EGTA, and 10 HEPES; pH was 7.8–8.0 after adding bicarbonate. A standard chloride solution contained the following (in mM): 130 KCl, 5 EGTA, and 10 HEPES; pH was adjusted to 7.8 with KOH. To record the HCO_3_^−^ selective conductance, the control bicarbonate solution was mixed with the standard chloride solution to make different concentrations (0, 16, 33, 65, and 130 mM) of HCO_3_^−^ around pH 7.8–8.0. To test the anion selectivity, KHCO_3_ in the control bicarbonate solution was replaced with anions such as KCl, K-gluconate, K-methanesulfonate, K-thiocyanate, or KI at pH 7.8. The concentration of free Ca^2+^ was calculated using the MaxChelator computer program. 4,4′-Diisothiocyano-2,2′-dihydrostilbenedisulfonic acid disodium salt (H_2_DIDS; Toronto Research Chemicals) was directly dissolved at 0.5 mM in control bicarbonate solution. 4-[[4-Oxo-2-thioxo-3-[3-trifluoromethyl)phenyl]-5-thiazolidinylidene]methyl]benzoic acid (CFTRinh-172; Santa Cruz Biotechnology) and 2-methyl-8-(phenylmethoxy)imidazo[1,2-a]pyridine-3-acetonitrile (Sch28080; Santa Cruz Biotechnology) were dissolved in DMSO at a 1000-fold concentration for application. The current was recorded in the inside-out configuration using the EPC 800 patch-clamp amplifier (HEKA). The amplifier was driven by Clampex 9 (Axon) in order to allow the delivery of a voltage-ramp protocol with concomitant digitization of the current. The membrane potential was generally held at 0 mV, and the command voltage was varied from − 80 to + 80 mV over a duration of 800 ms every 10 s.

### RT-PCR analysis

RNA was extracted from the interlobular (outside diameter of 50–150 μm) and main ducts (outside diameter of around 500 μm) from three independent guinea pigs using the RNeasy Plus Micro kit with DNase I (Qiagen). RT-PCR analysis was performed using the OneStep RT-PCR kit (Qiagen) with primers designed to recognize different types of transporters (Table 1). For the negative control of reverse transcription, we used *Taq* DNA Polymerase (Promega). The amplification parameters used were as follows: 1 cycle at 50 °C for 30 min and 1 cycle at 95 °C for 15 min, followed by 40 cycles at 94 °C for 30 s, 57 °C for 30 s, 72 °C for 30 s, and 1 cycle at 72 °C for 10 min. The transcripts were subsequently verified by agarose gel electrophoresis.

**Table 1:** Primer sets used for guinea pig pancreatic duct in RT-PCR analysis

Gene (subunit)		Size (bp)
Cftr		
Forward:	5′-GCTTAAAAGGACTATGGACACT -3′	623
Reverse:	5′-ACCTTCAGTGTTCAGCAGTCT -3′	
Gapdh		
Forward:	5′-CAAAAGGGTCATCATCTCTGC -3′	610
Reverse:	5′-GCCGAACTCATTGTCATACCA -3′	
CA2		
Forward:	5′-AGCCTCTGCACCTTCACTATG -3′	535
Reverse:	5′-AACATCTGCTCACTGCTTACG -3′	
Slc26a1		
Forward:	5′-CTACTCTGTCCGTGCCAACCA -3′	914
Reverse:	5′-ACAGCTGCTCATCCTCCATTC -3′	
Slc26a2		
Forward:	5′-GGGGTTGGTTTTTCTATGTTTTG -3′	554
Reverse:	5′-AAACCCACCGCTTCATACACG -3′	
Slc26a3		
Forward:	5′-GTATGAGCCAGAAGGAGTGAA -3′	459
Reverse:	5′-TACACATCTACATTTATCCTTGC -3′	
Slc26a4		
Forward:	5′-AAACATCCCCACCACAGACAT -3′	558
Reverse:	5′-AAACCACATTGCTCCATCTGC -3′	
Slc26a5		
Forward:	5′-GTGACCTTGCTCTCGGGAAT -3′	621
Reverse:	5′-GAAGAGGGAGCCGATGGAAT-3′	
Slc26a6		
Forward:	5′-TCGGTCCTCAGCCACTTTGTA -3′	544
Reverse:	5′-ATGCTGCTTGGTGATAGATGC-3′	
Slc26a7		
Forward:	5′-CCCCAATGAACCTCCTGTCTG-3′	713
Reverse:	5′-AAGTAGGTGATTAGTGGCATTC-3′	
Slc26a8		
Forward:	5′-TCGGGGCTTGGTCGTCTTG-3′	646
Reverse:	5′-AGGTTGATAGATGGGCTGGTA-3′	
Slc26a9		
Forward:	5′-CTATCTGTACCCTCTCCCTAA -3′	721
Reverse:	5′-AACGAGGGTATGGAAGGTAAC -3′	
Slc26a10		
Forward:	5′-ACTTTGCTGTGTGGATGGTCA-3′	550
Reverse:	5′-GCATCCTGGACACTCACAAAC-3′	
Slc26a11		
Forward:	5′-CAGGCAGCTTTGGGCGGAC-3′	566
Reverse:	5′-AGAGAAAACCAGGGAGACACC-3′	

### Immunolocalization

Immunolocalization was performed on the guinea pig pancreas. The pancreas was obtained from female Hartley guinea pigs (*n* = 3) in accordance with protocols approved by the Animal Experimentation Committee, Kansai Medical University. The guinea pigs were anesthetized with isoflurane and a mixture of medetomidine (0.5 mg/kg body weight), midazolam (5.0 mg/kg b.w.), and butorphanol (2.5 mg/kg b.w.), and perfused transcardially with 4% paraformaldehyde. The pancreas was fixed with 4% paraformaldehyde in PBS for 24 h, embedded in paraffin, and sectioned. Detailed methods for immunohistochemistry were described previously [12]. Briefly, autofluorescence was blocked by 0.1 M Tris-glycine. Non-specific binding was blocked with 2% normal donkey serum in PBS. Preparations were subsequently incubated with primary antibodies for SLC26A1, SLC26A3, SLC26A4, SLC26A6, or SLC26A10 (Table 2), along with Ezrin (1:400 to 1:800, clone 3C12, MS-661; Lab Vision) and PECAM-1 (platelet endothelial cell adhesion molecule-1, 1:800, sc-1506; Santa Cruz Biotechnology) in immunoreaction enhancer solution (Can Get Signal immunostain; Toyobo) overnight at 4 °C. Secondary antibodies conjugated to Alexa488 (SLC26A), Alexa568 (Ezrin), or Alexa647 (PECAM-1) (1:400; Molecular Probes) were added for 1 h. For the negative control, the primary antibodies were pre-absorbed with corresponding antigens for SLC26A1 (APrEST81987; Atlas), SLC26A10 (APrEST84901), SLC26A4 (synthesized peptide; Eurofins Genomics), or SLC26A6 (synthesized peptide) for 30 min at room temperature. In the controls, the primary antibodies were omitted and scanning was performed using the same settings. Nuclei were stained with 4′,6-diamidino-2-phenylindole (DAPI) at 1 μg/ml. Fluorescence was observed with a confocal laser scanning microscope (LSM510 META; Carl Zeiss).

**Table 2:** Antibodies used for guinea pig pancreatic duct in immunohistochemistry (IHC) and western blotting (WB)

Protein (accession)	Antigen	Correlation with guinea pig	Dilution		Catalogue number (manufacturer)
			IHC	WB	
SLC26A1 (NP_071325)	518–587	77%	1:100	1:500	HPA041654 (Atlas)
SLC26A3 (NP_000102)	617–733	85%	1:100	N/A	HPA036055 (Atlas)
SLC26A4 (NP_000432)	317–344	93%	1:200	1:1000	bs-6787R (Bioss antibodies)
SLC26A6 (NP_075062)	438–484	83%	1:200	1:1000	bs-20817R (Bioss antibodies)
SLC26A10 (NP_597996)	427–487	75%	1:200	1:100	HPA044719 (Atlas)

### Western immunoblotting

The pancreatic duct was dissected from three independent guinea pigs as described above. The ducts were washed with cold PBS, treated with trichloroacetic acid (10%) on ice for 30 min, and then centrifuged. The pellet was solubilized in lysis buffer containing urea (9 M), Triton X-100 (2%), dithiothreitol (1%), and lithium dodecyl sulfate (2%). The samples (30 μg/lane protein) were fractionated on SDS polyacrylamide gel (7.5%), electroblotted to PVDF membranes (Merck Millipore), blocked with skim milk (1%), and reacted with anti-SLC26A4, anti-SLC26A6, or anti-SLC26A10 antibodies (Table 2). For anti-SLC26A1, we used signal enhancer Hikari solution (Nacalai Tesque, Kyoto, Japan). The reaction was visualized with a secondary antibody labeled with alkaline phosphatase (Promega).

### Statistics

Data are shown as means ± SEM. A one-way analysis of variance (ANOVA) or Student’s paired *t* test was applied, and *P* < 0.05 was considered significant. Data were analyzed in Igor or Microsoft Excel.

## Results

### Bicarbonate conductance through the luminal membrane of the interlobular pancreatic duct cells

**Fig. 1:**
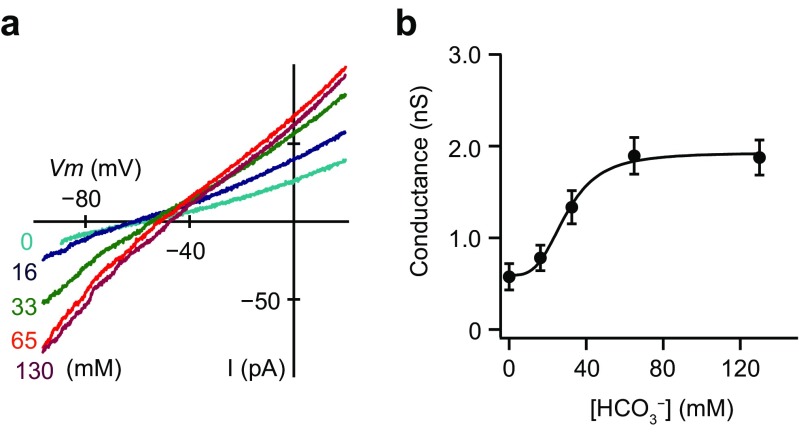
Bicarbonate conductance through the luminal membrane of the interlobular pancreatic duct cells. **a** Macroscopic current–voltage (*I*–*V*) relationships recorded from the luminal membrane of the pancreatic duct cells in the inside-out configuration with the standard NMDG-Cl pipette solution at different intracellular HCO_3_^−^ concentrations. Inward conductance attributed to HCO_3_^−^ efflux increased with HCO_3_^−^ concentration from 0 to 130 mM. **b** Linear plot of conductance by the HCO_3_^−^ concentration. The solid line is the fit by the Hill equation with the half-maximal effective concentration of 31.5 ± 5.1 mM and a Hill coefficient of 3.5 ± 0.4 (*n* = 5)

We recorded macroscopic currents from excised inside-out patches from the luminal membrane of the interlobular pancreatic duct cells of guinea pigs under unstimulated conditions. Figure 1a shows the macroscopic current–voltage (*I–V*) relationships in the presence of intracellular HCO_3_^−^ at different concentrations (0, 16, 33, 65, and 130 mM). As we used the standard NMDG-Cl pipette solution and the bathing solution containing KHCO_3_, the inward current was due to HCO_3_^−^ efflux through the luminal membrane. The inward conductance determined from the linear section of the *I–V* relationships (from − 80 to − 60 mV) increased with intracellular HCO_3_^−^ concentration (Fig. 1a). The linear plot of conductance with the HCO_3_^−^ concentration had a sigmoid relationship (Fig. 1b). The half-maximal effective concentration (K_d_) value for the effects of HCO_3_^−^ and Hill coefficient were 31.5 ± 5.1 mM and 3.5 ± 0.4 (*n* = 5), respectively. We also measured inward HCO_3_^−^ currents in the bathing solution containing 130 mM NaHCO_3_. The inward conductance increased to 2.04 ± 0.95 nS in NaHCO_3_ from 0.34 ± 0.08 nS in NaCl (data not shown; *n* = 13). Thus, there was a minor contribution of K^+^ conductance under unstimulated conditions.

### Ion selectivity of the bicarbonate conductance

**Fig. 2:**
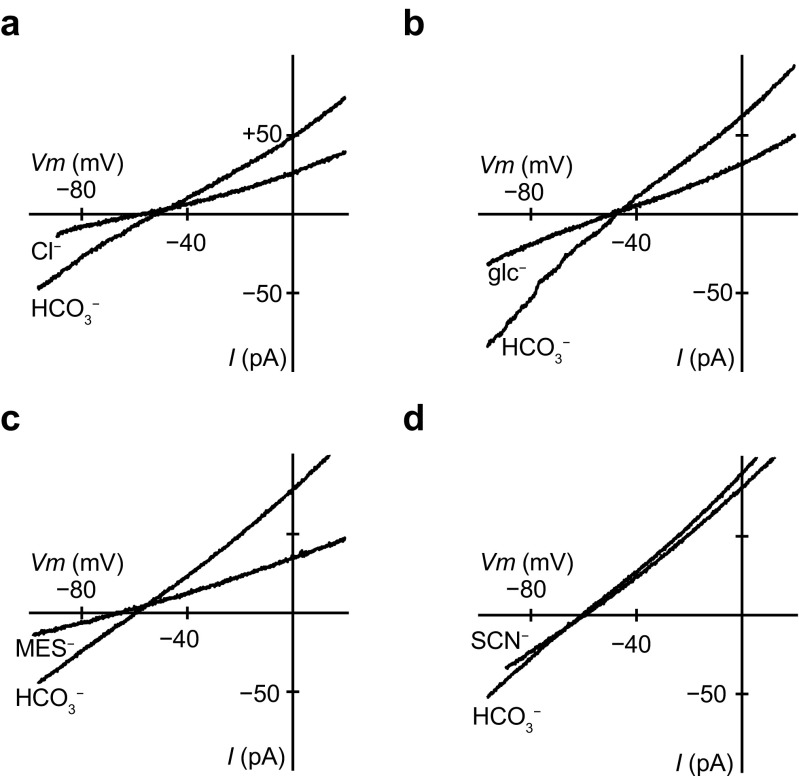
Ion selectivity of bicarbonate conductance. Macroscopic *I*–*V* relationships recorded from different inside-out patches. The intracellular 130 mM HCO_3_^−^ was substituted by equimolar chloride (Cl^−^), gluconate (glc^−^), methanesulfonate (MES^−^), or thiocyanate (SCN^−^) (*n* = 5–6). The inward conductance decreased significantly when HCO_3_^−^ was substituted with Cl^−^, glc^−^, or MES^−^

**Fig. 3:**
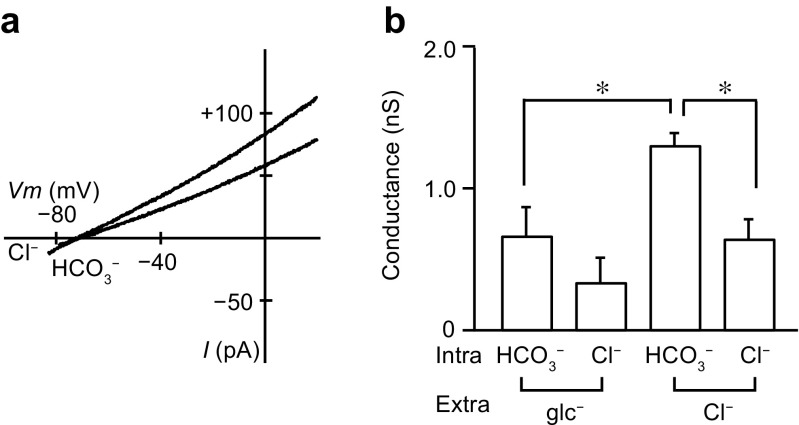
Effects of substitution of extracellular Cl^−^ with gluconate. **a** Macroscopic *I*–*V* relationships recorded from the interlobular pancreatic duct cells with gluconate-rich extracellular and 130 mM HCO_3_^−^ or 130 mM Cl^−^ intracellular solutions. **b** The inward conductance attributed to efflux of HCO_3_^−^ or Cl^−^ with gluconate-rich and standard NMDG-Cl pipette solutions (*n* = 5, *P < 0.05, ANOVA). Intra and Extra, intracellular and extracellular, respectively

Ion selectivity of the bicarbonate conductance was examined by replacing 130 mM HCO_3_^−^ in the intracellular bathing solution with other monovalent anions. Figure 2 shows macroscopic *I–V* relations recorded in the inside-out configuration with the standard NMDG-Cl pipette solution. In experiments where HCO_3_^−^ in the bath was replaced with Cl^−^ or gluconate (glc^−^), the reversal potential did not change, but inward conductance significantly decreased from 1.30 ± 0.09 nS in HCO_3_^−^ to 0.64 ± 0.13 nS in Cl^−^ (Figs. 2a and 3b (right); *n* = 5) and from 1.69 ± 0.08 nS in HCO_3_^−^ to 0.72 ± 0.14 nS in glc^−^ (Fig. 2b; *n* = 5). Replacement of HCO_3_^−^ with methanesulfonate (MES^−^) shifted the reversal potential in a negative direction, indicating it was less permeable than HCO_3_^−^, and the inward conductance significantly decreased from 2.56 ± 0.73 nS in HCO_3_^−^ to 0.73 ± 0.36 nS in MES^−^ (Fig. 2c; *n* = 6). Replacement of HCO_3_^−^ with thiocyanate (SCN^−^) slightly shifted the reversal potential in a positive direction, but the inward conductance had little change (Fig. 2d; *n* = 6). Finally, replacement of HCO_3_^−^ with iodide (I^−^) did not cause a marked difference in the reversal potential or the inward conductance (data not shown; *n* = 6). We calculated the permeability ratio (*P*_X_/*P*_HCO3_) from the shift in the reversal potential (Δ*V*_rev_) when anion X is substituted for internal HCO_3_^−^ [13]; that is, from:

$$ \Delta {V}_{\mathrm{rev}}=\left(\mathrm{RT}/\mathrm{F}\right)\times \ln \left({P}_{\mathrm{x}}{\left[{\mathrm{X}}^{-}\right]}_{\mathrm{i}}/{P}_{\mathrm{HCO}3}{\left[{{\mathrm{HCO}}_3}^{-}\right]}_{\mathrm{i}}\right), $$

where R, T, and F have their conventional thermodynamic meanings. The sequence of the permeability ratios was SCN^−^ (1.41 ± 0.15) > Cl^−^ (1.18 ± 0.14) = glc^−^ (1.07 ± 0.03) = I^−^ (1.06 ± 0.06) = HCO_3_^−^ (1.00) > MES^−^ (0.65 ± 0.11) (*n* = 5–6). The sequence of the relative inward conductance determined from − 80 to − 60 mV was HCO_3_^−^ (1.00) > SCN^−^ (0.69 ± 0.10) = I^−^ (0.66 ± 0.09) > Cl^−^ (0.48 ± 0.08) = glc^−^ (0.43 ± 0.09) > MES^−^ (0.26 ± 0.06) (*n* = 5–6).

### Bicarbonate conductance is dependent on luminal Cl^−^

To evaluate the activities of Cl^−^–HCO_3_^−^ exchangers on the apical membrane of interlobular pancreatic ducts of the guinea pig, Ishiguro and colleagues replaced Cl^−^ with gluconate in the lumen [14, 16, 43]. Similarly, we recorded macroscopic currents with extracellular solution containing 120 mM gluconate and 10 mM Cl^−^. With the control intracellular solution_,_ *E*_rev_ was − 46.6 ± 4.5 mV with a standard NMDG-Cl pipette solution (Fig. 2a; *n* = 5) and − 63.6 ± 3.9 mV with gluconate-rich pipette solution (Fig. 3a; *n* = 5), demonstrating a significant difference (ANOVA). We also compared the inward HCO_3_^−^ conductance with gluconate-rich and standard NMDG-Cl pipette solutions (Fig. 3b). The HCO_3_^−^ conductance was significantly lower with the gluconate-rich pipette solution (0.66 ± 0.20 nS) than with standard NMDG-Cl pipette solutions (1.30 ± 0.09 nS) (*n* = 5, ANOVA). Additionally, as described in the previous section, the inward conductance significantly decreased when HCO_3_^−^ in the bath was replaced with Cl^−^, indicating that there was a minor contribution from Cl^−^-dependent current (Fig. 3b, right). However, the inward conductance was not significantly different with gluconate-rich pipette solution (Fig. 3b, left). The results described so far indicate that both intracellular HCO_3_^−^ and luminal Cl^−^ are essential for the HCO_3_^−^ conductance, and that the HCO_3_^−^ conductance is carried out through Cl^−^–HCO_3_^−^ exchangers on the luminal membrane.

### Effects of the anion exchanger inhibitor H_2_DIDS on bicarbonate conductance

**Fig. 4:**
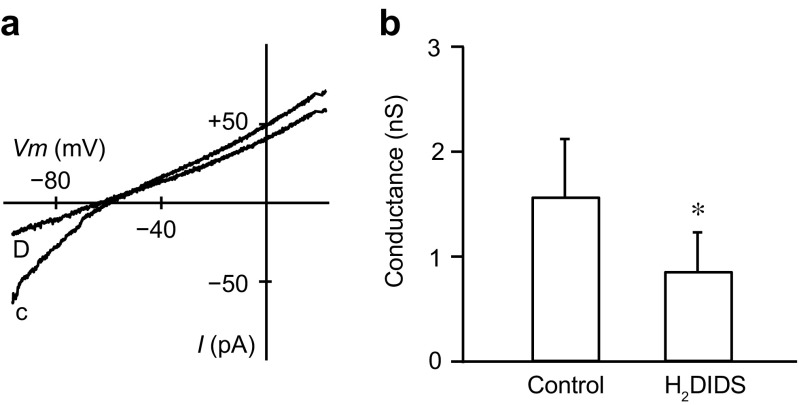
Effects of H_2_DIDS on bicarbonate conductance. **a** Macroscopic *I*–*V* relationships obtained in the absence or presence of 0.5 mM intracellular H_2_DIDS. c, control; D, H_2_DIDS. CFTRinh-172 at 20 μM was added to the standard NMDG-Cl pipette solution. **b** H_2_DIDS significantly decreased the average inward HCO_3_^−^ conductance (*n* = 6, *P < 0.05)

Previous studies reported that Cl^−^–HCO_3_^−^ exchangers were inhibited by luminal H_2_DIDS, a disulfonic stilbene [14, 43]. For experimental ease, we applied H_2_DIDS (0.5 mM) intracellularly while recording macroscopic currents from excised inside-out patches with the control bicarbonate internal and the standard NMDG-Cl pipette solutions. To exclude the possibility of the contamination of CFTR Cl^−^ conductance, we included 20 μM CFTRinh-172, an inhibitor of CFTR Cl^−^ channels, in the pipette solution. H_2_DIDS applied intracellularly significantly decreased inward HCO_3_^−^ conductance from 1.57 ± 0.55 to 0.86 ± 0.37 nS (Fig. 4; *n* = 6). We also tested 30 μM Sch28080, a H^+^–K^+^-pump inhibitor, but did not observe any inhibitory effects on the inward HCO_3_^−^ conductance (*n* = 4; not shown). These results further support that the HCO_3_^−^ conductance occurs through Cl^−^–HCO_3_^−^ exchangers.

### Regulation of bicarbonate conductance by intracellular ATP and cAMP

**Fig. 5:**
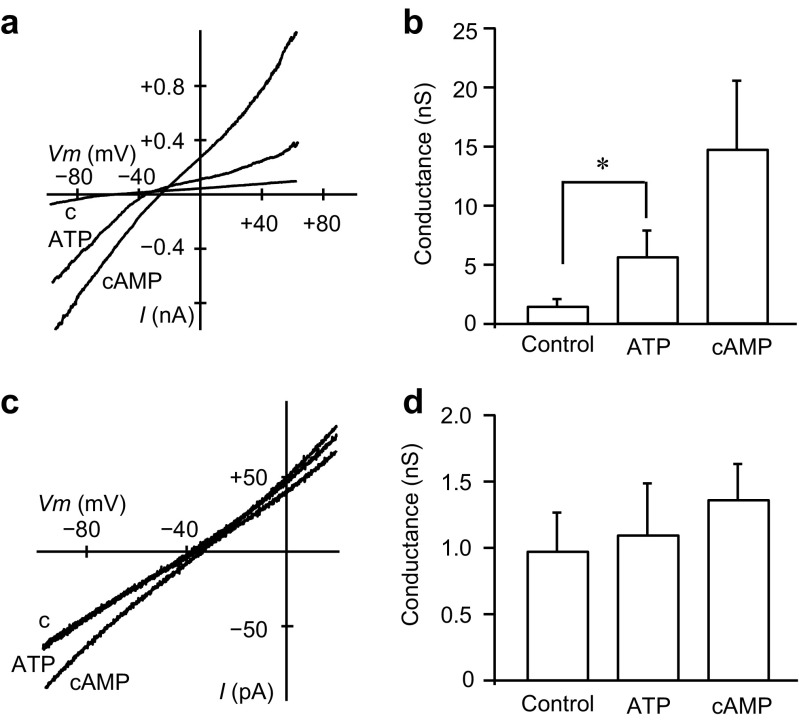
Activation of bicarbonate conductance by intracellular ATP and cAMP. **a** Macroscopic *I*–*V* relationships from the interlobular pancreatic duct cells with the control bicarbonate internal solution (c), and with addition of ATP alone, or ATP and cAMP. The standard NMDG-Cl pipette solution was used. **b** Averaged HCO_3_^−^ conductance with the control, ATP alone (*n* = 13, *P < 0.05), or ATP + cAMP (*n* = 4). **c** Macroscopic *I*–*V* relationships obtained in the presence of extracellular CFTRinh-172 at 20 μM along with the standard NMDG-Cl pipette solution. **d** Averaged HCO_3_^−^ conductance with the control, ATP alone, or ATP + cAMP (*n* = 11)

In pancreatic duct cells, cAMP and Ca^2+^ signaling pathways play a role in fluid secretion. As CFTR Cl^−^ channels were regulated by intracellular cAMP [6, 8, 29] and ATP [40], we tested their effects on bicarbonate conductance. Application of intracellular 2 mM ATP-Mg significantly increased the inward conductance from 1.51 ± 0.59 to 5.70 ± 2.18 nS (Fig. 5a, b; *n* = 13). The addition of 1 mM cAMP further increased the inward conductance to 14.8 ± 5.57 nS (*n* = 4). cAMP also activated the marked outward conductance, which was attributed to Cl^−^ influx, most likely through CFTR Cl^−^ channels. Therefore, we tested the effects of intracellular ATP-Mg and cAMP with the pipette solution including CFTRinh-172 at 20 μM. In the presence of CFTRinh-172, application of intracellular 2 mM ATP-Mg and 1 mM cAMP had little effect on the conductance in either direction (Fig. 5c): the inward conductance was not significantly increased in the presence of ATP-Mg (1.11 ± 0.39 nS) or cAMP (1.37 ± 0.27 nS) as compared with the control (0.98 ± 0.29 nS) (Fig. 5d; *n* = 11). These results indicate that intracellular ATP and cAMP may not directly regulate Cl^−^–HCO_3_^−^ exchangers, but instead regulate CFTR Cl^−^ channels on the luminal membrane of duct cells. Additionally, 1 μM free Ca^2+^ added to the intracellular solution did not affect the inward HCO_3_^−^ conductance (*n* = 3; not shown), suggesting that intracellular Ca^2+^ does not regulate Cl^−^–HCO_3_^−^ exchangers directly.

### Pancreatic duct epithelia expressed a variety of SLC26A family members

**Fig. 6:**
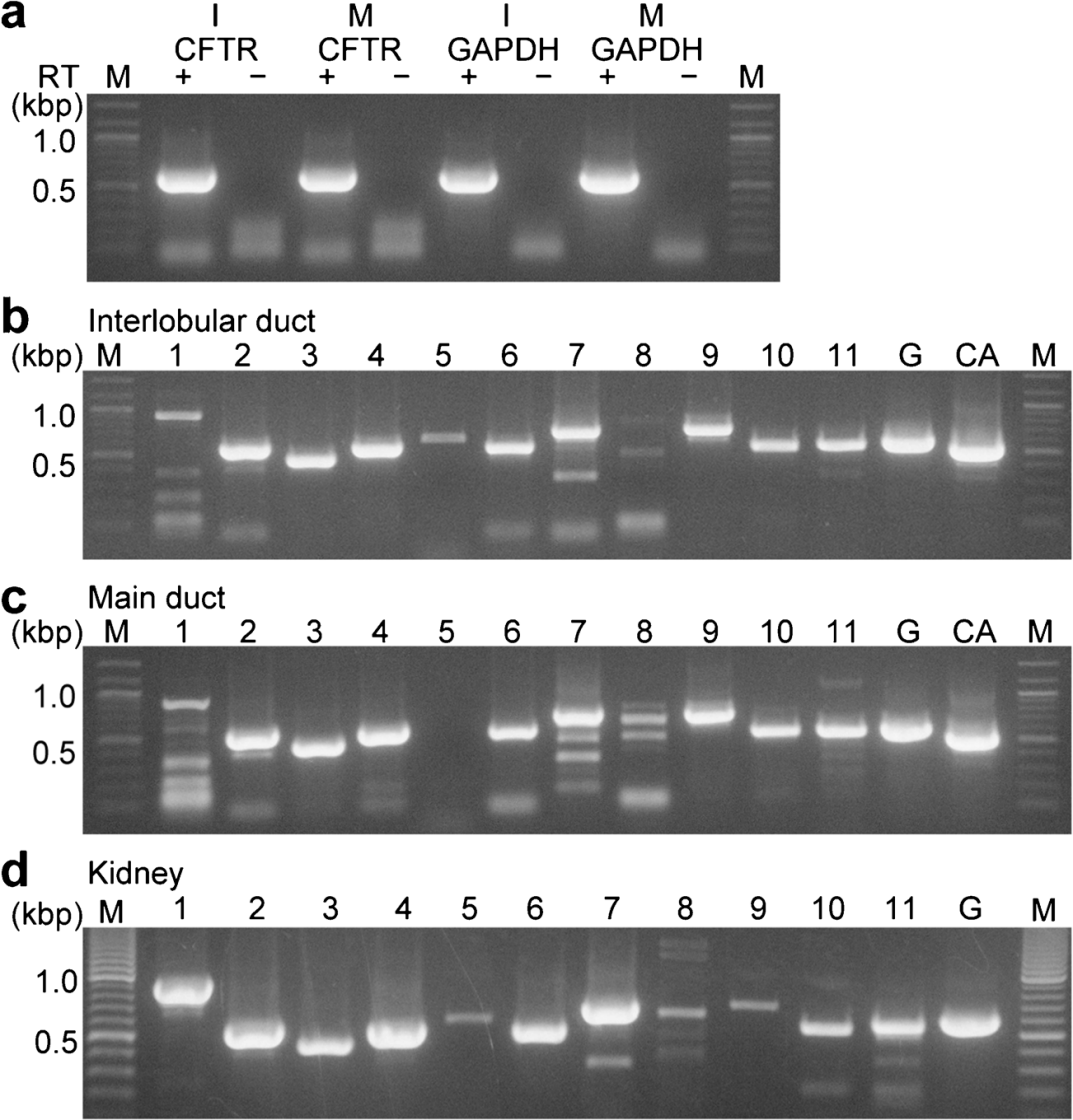
RT-PCR analysis of the SLC26A family. Ethidium bromide-stained agarose gels show RT-PCR products generated from total RNA isolated from the interlobular (I) and main (M) pancreatic ducts. **a** Control experiment shows the amplification of Cftr (623 bp) and Gapdh (610 bp). No DNA fragment was amplified with the template without reverse transcription (RT). The primers for the RT-PCR analysis from the interlobular (**b**) and main (**c**) ducts gave the expected fragment length for *Slc26a1–11* (Table 1). **d** Positive control obtained from the kidney. A representative gel for at least three independent experiments is shown. M in **a**–**d**: molecular mass, G in **b**–**d**: GAPDH, CA in **b** and **c**: carbonic anhydrase II

It is known that anion exchangers in pancreatic duct cells are members of the SLC26A family [26]. Two members of the family, SLC26A3 (DRA; downregulated in adenoma) [25] and SLC26A6 (PAT1; putative anion transporter-1) [24, 46], were reported to be expressed in the luminal membrane of pancreatic ducts and function as Cl^−^–HCO_3_^−^ exchangers [9, 19, 20]. Interlobular ducts from guinea pigs expressed mRNAs encoding *Slc26a3* and *Slc26a6* [43]. In the present study, we evaluated the expression of all members of the SLC26A family using RT-PCR analysis on isolated interlobular and main ducts. Figure 6a shows the isolated interlobular and main pancreatic ducts expressing CFTR and GAPDH. Then, we screened all 11 members of the SLC26A family from the interlobular ducts (Fig. 6b; *n* = 3 animals) and main ducts (Fig. 6c; *n* = 3 animals), along with GAPDH and a duct marker of carbonic anhydrase II (CA2). We also screened all primer sets from the total RNA of the kidney as a positive control (Fig. 6d). RT-PCR analysis revealed that the interlobular and main ducts expressed *Slc26a1*, *Slc26a2*, *Slc26a3*, *Slc26a4*, *Slc26a6*, *Slc26a7*, *Slc26a9*, *Slc26a10*, and *Slc26a11*.

### Immunolocalization of the SLC26A family in pancreatic duct cells

**Fig. 7:**
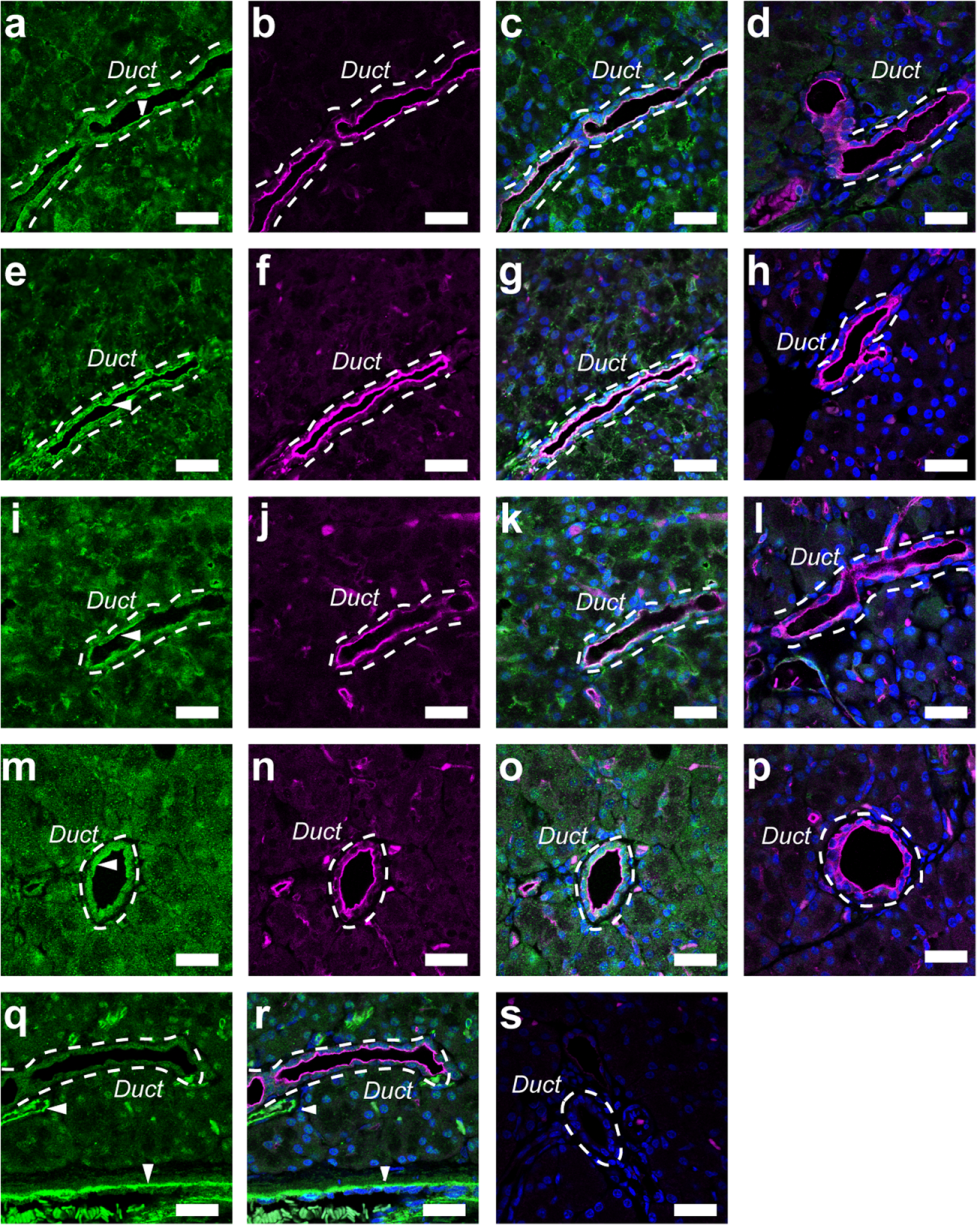
Immunolocalization of the SLC26A family in the interlobular pancreatic duct. **a** Fluorescence of SLC26A6 on the luminal membranes of duct cells. **b** Fluorescence image of ezrin. **c** Overlay image of **a** and **b**. **d** Overlay image of ezrin and green fluorescence with SLC26A6 antibody pre-absorbed with the corresponding antigen. The *broken line* indicates a duct. *Arrowhead* indicates the primary antibody signal on the luminal membrane. Fluorescence images of SLC26A1 (**e**), ezrin (**f**), overlay (**g**), and negative control with pre-absorbed SLC26A1 antibody (**h**). Fluorescence image of SLC26A4 (**i**), ezrin (**j**), overlay (**k**), and negative control with pre-absorbed SLC26A4 antibody (**l**). Fluorescence images of SLC26A10 (**m**), ezrin (**n**), overlay (**o**), and negative control with pre-absorbed SLC26A10 antibody (**p**). Fluorescence images of PECAM-1 (**q**), a blood vessel marker, and overlay with ezrin (**r**). *Arrowheads* show a blood vessel that does not overlap with the duct. **s** Control image of the guinea pig pancreas, in which primary antibodies were omitted. DAPI was used to stain nuclei (*blue*). Representative images for at least three independent experiments are shown. *Bars* = 20 μm

The immunolocalization of the SLC26A family was examined with paraffin sections of guinea pig pancreas. Immunofluorescence ascribed to the SLC26A exchanger was colocalized with Ezrin, an A-kinase anchoring protein, to the luminal membrane of the pancreatic duct (Fig. 7). In the guinea pig pancreas, immunofluorescence of SLC26A6 was detected on the luminal membranes of duct cells (Fig. 7a), as reported for the rat pancreas previously [20]. SLC26A6 were colocalized with Ezrin to the luminal membranes (Fig. 7b, c). The immunofluorescence on the luminal membranes was diminished with SLC26A6 antibody, which was pre-absorbed with the corresponding antigen for the negative control (Fig. 7d). Additionally, a strong signal ascribed to SLC26A1 was detected and colocalized with Ezrin to the luminal membrane (Fig. 7e–g). We also detected immunofluorescence of SLC26A4 and SLC26A10 on the luminal membrane of the duct cells (Fig. 7i–k and m–o, respectively). The immunofluorescence was reduced when antibodies were pre-absorbed with the corresponding antigens (Fig. 7h, l, p). We used HPA036055 (Atlas) as the anti-SLC26A3 antibody, but failed to immunostain SLC26A3 in the guinea pig pancreas. We stained with PECAM-1, a blood vessel marker, to distinguish between pancreatic ducts and blood vessels (Fig. 7q, r).

### Expression of SLC26A protein in guinea pig pancreatic ducts

**Fig. 8:**
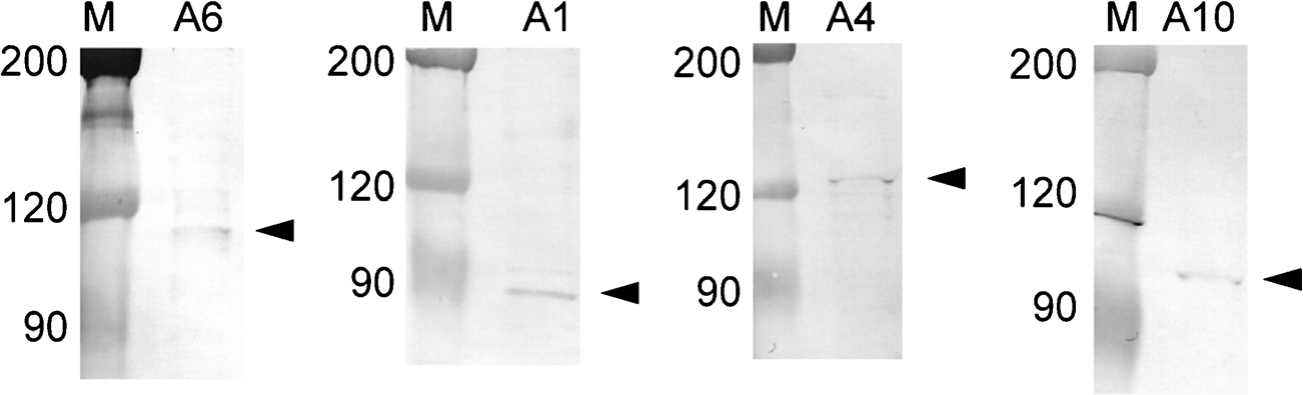
Immunoblot of the SLC26A family from the pancreatic duct. Protein samples were resolved by SDS-PAGE. *Arrowheads* indicate SLC26A proteins detected by immunoblotting using anti-SLC26A antibodies. Representative membranes for at least three independent experiments are shown. M, marker; A6, SLC26A6; A1, SLC26A1; A4, SLC26A4; A10, SLC26A10

We next performed western blot analysis to examine the expression of SLC26A protein in the guinea pig pancreatic ducts. We detected SLC26A6 (~ 107 kDa), SLC26A1 (~ 78 kDa), SLC26A4 (~ 136 kDa), and SLC26A10 (~ 108 kDa) in the lysates of the isolated ducts (Fig. 8; *n* = 3 animals). The molecular mass values corresponded to those of human SLC26A proteins (~ 100 kDa), which were *N*-glycosylated, expressed in HEK-293 cells [22].

## Discussion

In the present study, we applied patch electrodes on the luminal membrane of guinea pig pancreatic duct cells and recorded macroscopic currents in the inside-out configuration. The inward conductance was dependent on the intracellular HCO_3_^−^ concentration (Fig. 1) and was reduced when intracellular HCO_3_^−^ was replaced with Cl^−^, glc^−^, or MES^−^ (Fig. 2) or extracellular Cl^−^ was replaced with glc^−^ (Fig. 3). Furthermore, the inward conductance was decreased in the presence of H_2_DIDS, an inhibitor of Cl^−^–HCO_3_^−^ exchangers (Fig. 4). These electrophysiological findings suggested that the inward conductance was ascribed to HCO_3_^−^ efflux through Cl^−^–HCO_3_^−^ exchangers on the luminal membrane. In addition, we found that SLC26A1, SLC26A4, SLC26A6, and SLC26A10 were localized to the luminal membrane of the pancreatic duct cells (Figs. 7 and 8).

HCO_3_^−^ can flow outwardly not only through Cl^−^–HCO_3_^−^ exchangers but also through CFTR Cl^−^ channels on the luminal membrane [7]. The permeability ratio sequence of the Cl^−^ channel in inside-out patches from the rat pancreatic duct cells was NO_3_^−^ > Cl^−^ > HCO_3_^−^ > gluconate [7], and that in whole-cell patches from the guinea pig pancreatic duct cells was Br^−^ > I^−^ = Cl^−^ > HCO_3_^−^ > ClO_4_^−^ > aspartate [29]. These were different from the permeability ratio sequence of the inward conductance obtained in the present study: SCN^−^ > Cl^−^ = gluconate = I^−^ = HCO_3_^−^ > MES^−^ (Fig. 2). Similarly, a previous study reported that the anion selectivity of SLC26A6 in HEK 293 cells was SCN^−^ > NO_3_^−^ > Cl^−^ [30]. Single-channel and whole-cell conductance through Cl^−^ channels was reduced in the presence of HCO_3_^−^ [7, 29], whereas the inward conductance was increased with increasing intracellular HCO_3_^−^ in our experiments (Fig. 1). These results suggest that HCO_3_^−^ efflux occurs by pathway independent from the Cl^−^ channels. We followed previous studies that evaluated the activities of Cl^−^–HCO_3_^−^ exchangers on the apical membrane of pancreatic ducts by replacing extracellular Cl^−^ with gluconate [14, 16, 43], and observed that the reversal potential shifted to a negative direction and the inward HCO_3_^−^ conductance decreased (Fig. 3). The dependency of inward HCO_3_^−^ conductance on extracellular Cl^−^ suggests that HCO_3_^−^ is exchanged for Cl^−^. Our results demonstrated that intracellular HCO_3_^−^ increased the conductance with a K_d_ value of approximately 30 mM (Fig. 1), corresponding to the physiological concentration of intracellular HCO_3_^−^ in duct cells. Additionally, the Hill coefficient, which was estimated to be 3.5 for the effects of HCO_3_^−^, suggested that positive cooperative binding of HCO_3_^−^ facilitated the binding of subsequent HCO_3_^−^ at other sites on Cl^−^–HCO_3_^−^ exchangers.

We detected SLC26A1, SLC26A4, SLC26A6, and SLC26A10 on the luminal membrane of the interlobular pancreatic duct (Fig. 7). SLC26A6 was localized to the luminal membrane of interlobular pancreatic ducts of humans [24] and rats [20], as well as to the intestine, kidney, parotid gland, and heart [1, 20, 21, 24, 47]. SLC26A6 cloned from guinea pig pancreatic ducts mediated Cl^−^–HCO_3_^−^ exchange in HEK 293 cells [44]. SLC26A4 (pendrin) was localized to the apical membranes of the submandibular duct, type B and non-A, non-B intercalated cells in the cortical collecting duct of the kidney, and thyroid follicular cells, and was expressed in inner ear [3, 36, 37, 39]. SLC26A4 mediates HCO_3_^−^ secretion across the apical membrane in Calu-3, a human airway epithelia cell line, monolayers [5] and in the cortical collecting ducts [37]. SLC26A1 identified as sulfate/bicarbonate/oxalate exchangers was expressed in the liver and kidney, and to a lesser extent, in the pancreas and testis [2, 35], and detected on the basolateral membrane of kidney and liver epithelial cells [18, 34]. SLC26A10 was found at the mRNA level in the heart and sarcoma [1, 4], but its function is unknown. Although the previous study demonstrated localization of SLC26A3 to the apical membrane of mouse pancreatic duct cells [9], we were unable to immunostain SLC26A3 in guinea pig pancreatic duct cells. The immunostaining signal in the guinea pig pancreas may be underestimated because we were only able to use antibodies against the human SLC26A family. Future studies are needed to establish the functional relevance of SLC26A molecules in pancreatic ducts.

We found that intracellular ATP and cAMP activated anion conductance on the luminal membrane in guinea pig pancreatic duct cells (Fig. 5a, b), as observed in rat pancreatic duct cells [6]. It was reported using HEK293 cells that CFTR stimulated by forskolin activated anion exchange of SLC26A3, SLC26A4, and SLC26A6 [19]. Thus, the increased conductance was attributed to activation of CFTR Cl^−^ channels by intracellular ATP and cAMP [6, 8, 29, 40], and activation of Cl^−^–HCO_3_^−^ exchangers by activated CFTR [19]. As anion conductance was not significantly increased in the presence of CFTRinh-172 in the pipette solution (Fig. 5c, d), we concluded that intracellular ATP and cAMP may not directly regulate Cl^−^–HCO_3_^−^ exchangers.

We found that H_2_DIDS applied intracellularly inhibited inward HCO_3_^−^ conductance by 50% in excised inside-out patches from the luminal membrane (Fig. 4). A previous study demonstrated that other disulfonic stilbenes, 4,4′-dinitrostilbene-2,2′-disulphonic acid and 4,4′-diisothiocyanostilbene-2,2′-disulfonic acid, blocked CFTR Cl^−^ channels when applied to the cytoplasmic face of membrane patches, with K_d_ values (at 0 mV) of 160 and 80 μM, respectively [23]. It is likely that disulfonic stilbenes are able to act on Cl^−^–HCO_3_^−^ exchangers from not only the outside but also from the inside of the cell membrane.

**Fig. 9:**
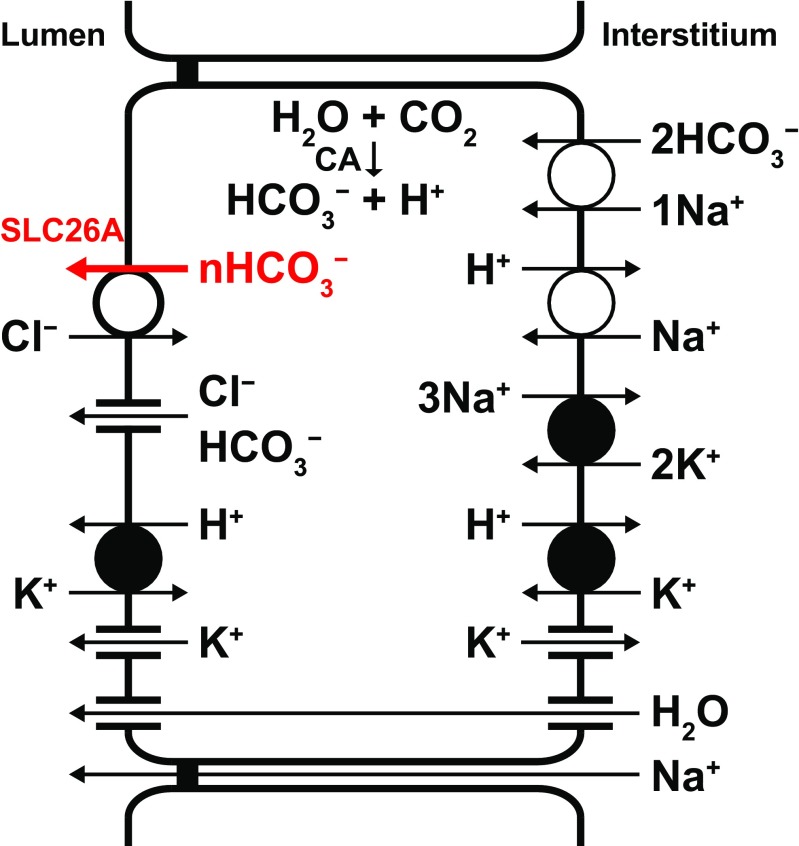
Model of HCO_3_^−^ transport in a pancreatic duct cell. Intracellular HCO_3_^−^ is derived from CO_2_ through the action of carbonic anhydrase (CA) and from HCO_3_^−^ uptake via the Na^+^–HCO_3_^−^ cotransporter. H^+^ is extruded at the basolateral membrane by the Na^+^–H^+^ exchanger and H^+^–K^+^ pump. HCO_3_^−^ efflux across the luminal membrane is mediated by Cl^−^ channels (CFTR and TMEM16A/ANO1) and electrogenic Cl^−^–nHCO_3_^−^ exchangers (SLC26A1, 4, 6, and/or 10; *n* > 1). K^+^ channels provide an exit pathway for K^+^ and play a vital role in maintaining the membrane potential, which is a crucial component of the driving force for anion secretion. Luminal H^+^–K^+^ pumps may provide a buffering/protection zone for the alkali-secreting epithelium. Primary active transport is indicated by filled circles

In conclusion, we used the patch-clamp technique in the inside-out configuration and demonstrated that the HCO_3_^−^ conductance through the luminal membrane is mediated by Cl^−^–HCO_3_^−^ exchangers under physiological HCO_3_^−^ concentrations in pancreatic duct cells. Our findings suggest that SLC26A1, SLC26A4, SLC26A6, and SLC26A10 may be involved in the HCO_3_^−^ transport through the luminal membrane. The SLC26A family may also play a role in pH homeostasis in the pancreatic lumen and duct cells. The direct measurement of the HCO_3_^−^ current from the interlobular duct and its functional characterization helps to propose a useful model for HCO_3_^−^ secretion from the pancreatic duct epithelia (Fig. 9).
